# Sacred Moment Experiences Among Internal Medicine Physicians

**DOI:** 10.1001/jamanetworkopen.2025.13159

**Published:** 2025-05-30

**Authors:** Jessica Ameling, Nathan Houchens, M. Todd Greene, David Ratz, Martha Quinn, Latoya Kuhn, Sanjay Saint

**Affiliations:** 1 Department of Internal Medicine, University of Michigan Medical School, Ann Arbor; 2 Medicine Service, Veterans Affairs Ann Arbor Healthcare System, Ann Arbor, Michigan; 3 Center for Clinical Management Research, Veterans Affairs Ann Arbor Healthcare System, Ann Arbor, Michigan; 4 University of Michigan School of Public Health, Ann Arbor

## Abstract

**Importance:**

Sacred moments—meaningful, memorable, and sometimes spiritual moments of connection—occur in health care and may enhance clinician well-being.

**Objective:**

To assess the prevalence, associated factors, and potential benefits of sacred moments among physicians.

**Design, Setting, and Participants:**

This cross-sectional national survey of randomly selected US internal medicine physicians with no subspecialties was conducted from June 23, 2023, to May 8, 2024. Participants were identified from a national physician database. Surveys assessed physician characteristics, burnout (defined as an occupational phenomenon of emotional exhaustion, cynicism, depersonalization, and a sense of low personal accomplishment), and factors hypothesized to be protective against burnout, including the occurrence and frequency of experiencing sacred moments with patients. Religious or spiritual beliefs, interpersonal connections, and sense of purpose in life and work were also assessed.

**Exposure:**

The Internist Wellness Survey.

**Main Outcomes and Measures:**

Three domains of burnout, including emotional exhaustion, depersonalization, and reduced personal accomplishment, were assessed using the Maslach Burnout Inventory–Human Services Survey. Multivariable logistic regression models assessed associations between physician characteristics and sacred moments and the association between sacred moments and burnout.

**Results:**

Of 1421 randomly selected internal medicine physicians, 629 completed a survey (44.3% response rate). Physicians spent a median of 23 (IQR, 15-29) years practicing; 376 of 617 (60.9%) were male. A total of 299 of 617 respondents (48.5 %) were hospitalists while 307 of 618 (49.7%) were primary care providers. A total of 424 of 626 respondents (67.7%) reported experiencing a sacred moment with a patient. Of the 421 respondents supplying additional responses, 19 (4.5%) often or always discussed these experiences with colleagues. Factors associated with the greatest increased odds of experiencing a sacred moment included considering oneself a very spiritual person (odds ratio [OR], 2.23; 95% CI, 1.44-3.44; *P* < .001) and having a strong sense of purpose in life (OR, 1.94; 95% CI, 1.36-2.76; *P* < .001) and work (OR, 1.94; 95% CI, 1.35-2.79; *P* < .001). Compared with those experiencing sacred moments less often, respondents experiencing sacred moments a few times per year or more frequently had reduced odds of extreme burnout (OR, 0.29; 95% CI, 0.14-0.60; *P* = .001). Discussing sacred moments with colleagues was associated with lower odds of burnout (OR, 0.62; 95% CI, 0.40-0.95; *P* = .03).

**Conclusions and Relevance:**

In this national survey of internal medicine physicians, experiencing sacred moments is common; discussing them with colleagues is not. These findings suggest that frequently experiencing sacred moments and discussing them with colleagues could bolster physician well-being.

## Key Points

**Question:**

What are the prevalence, associated factors, and potential benefits of sacred moments—brief periods of meaningful connection between clinician and patient—among US internal medicine physicians?

**Findings:**

In this survey study of 629 physicians, 68% reported experiencing a sacred moment with a patient, and physicians who considered themselves very spiritual or possessing a strong sense of purpose in life or work had increased odds of experiencing a sacred moment. Both experiencing sacred moments often and discussing them with colleagues were associated with less burnout.

**Meaning:**

These findings suggest that fostering sacred moment experiences and discussing them with others could help improve physician well-being.

## Teaser

This survey study measures the prevalence of experiencing sacred moments, how often they were discussed with colleagues, and their association with burnout among internal medicine physicians.

## Introduction

Sacred moments are short periods of time that evoke powerful emotions and/or spiritual qualities of transcendence and boundlessness, as if time stood still.^1,2^ Some have described sacred moments as sudden intimacies or moments of deep memorable connection with another person.^2,3^ The health care setting is replete with opportunities for the types of human connections that lead to sacred moments, such as times of crises or grief, or conversely, times of great happiness.^3^ These moments leave participants with a sense of joy, peace, and empathy for the others involved.^2^

Qualitative work with physicians, nurses, and patients suggest that sacred moments may be quite common and beneficial to both patients and clinicians.^2,4^ This is noteworthy in the context of physician burnout—defined as an occupational phenomenon of emotional exhaustion, cynicism, depersonalization, and a sense of low personal accomplishment—with rates that have been reported as high as 63% and nursing burnout rates as high as 47%.^5,6^ Sacred moment experiences have been described as a buffer against clinician burnout and “the reason I went into medicine.”^2^ They may represent a novel approach to addressing physician burnout. Several studies in general community and psychotherapy settings have identified factors associated with perceptions of sacred moments, including religious, spiritual, or supernatural beliefs and deep interpersonal connections.^7,8^ However, the prevalence of sacred moments, factors associated with experiencing sacred moments, and potential benefits of sacred moments on manifestations of burnout have not to our knowledge been studied in a national physician sample.

We sought to understand internal medicine physicians’ experiences with sacred moments. Specifically, we measured the prevalence of experiencing sacred moments and how often these experiences were discussed among internal medicine physicians. We also identified factors associated with experiencing sacred moments and potential benefits of these moments in burnout manifestations.

## Methods

### Study Design and Data Collection

We conducted a cross-sectional survey of US internal medicine physicians between June 23, 2023, and May 8, 2024. The overall goal of the survey was to assess physician well-being and burnout. We also measured factors hypothesized to either protect against or contribute to burnout, including experiencing sacred moments.

Our national cohort of internal medicine physicians was selected randomly from Physician Professional Data, a national physician database maintained by the American Medical Association. We obtained physician names and street addresses through a commercial third-party vendor (Redi-Data; Redi-Data Inc). We oversampled hospitalists and Veterans Affairs physicians, and the remaining sample was evenly split among the 4 major US geographic regions. Our cohort was selected from internal medicine physicians without a subspecialty. Selected physicians were mailed a paper copy of the survey with a US $20 cash incentive included. The invitation also included a link to complete an electronic version of the survey online if they preferred. Several reminders and additional survey mailings were provided to maximize response rate. This study was approved as exempt research without required informed consent by the University of Michigan Institutional Review Board under 45 CFR 46 Exemption 2 and follows the American Association for Public Opinion Research (AAPOR) reporting guideline for survey studies.

### Study Measures

The complete survey consisted of 42 questions. Sacred moment prevalence was measured by 2 questions asking physicians if they ever experienced a sacred moment with a patient (yes or no), and, if yes, how often they experience sacred moments. Sacred moments were defined on the survey as “deeply memorable, and sometimes spiritual moments shared between physicians and patients—as if time stood still. These may happen spontaneously during times of crisis or sadness, or conversely during times of great joy.” If respondents answered “yes” to experiencing a sacred moment, they were also asked how often they discuss these experiences with colleagues. The final sacred moment question asked respondents to indicate, using a Likert scale from 1 to 5, the extent to which they agree or disagree with the following statement: “Experiencing a sacred moment with a patient helps me feel less burned out.”

We measured several factors we hypothesized to be independently associated with having had a sacred moment with a patient: religious or spiritual beliefs, interpersonal connections, and perceived sense of purpose in life and work. We measured manifestations of burnout using the well-validated 22-item Maslach Burnout Inventory–Human Services Survey (under license from Mind Garden Inc),^9^ which assesses 3 domains of burnout: emotional exhaustion, depersonalization, and reduced personal accomplishment. Consistent with prior research,^10^ a dichotomous outcome of at least 1 manifestation of burnout was defined as scoring high in the emotional exhaustion (ie, ≥27) and/or depersonalization (ie, ≥10) domains. Given a lack of consensus on defining burnout, we also used another common dichotomous outcome of extreme burnout defined as scores meeting set thresholds in all 3 burnout domains (ie, emotional exhaustion, ≥27; depersonalization, ≥10; and personal accomplishment, ≤33).^10^ Finally, we collected self-reported data on physician demographic characteristics, including gender, race and ethnicity, marital and family status, time in practice, and type, setting, and context of clinical care. Respondents self-reported race and ethnicity as American Indian or Alaska Native, Asian, Black or African American, Hispanic or Latino, Native Hawaiian or Other Pacific Islander, White, or other (written responses included Afghan, Arab, Asian Indian, Egyptian, human, Indian, Iranian, Mediterranean, Mestizo, Middle Eastern, Middle Eastern North African, Pakistani, Persian, Portuguese-African, South Asian, South Indian, Southeast Asian, and multiracial). Race and ethnicity data were collected as we hypothesized these factors to be related to both sacred moments and burnout. 

### Statistical Analysis

Descriptive statistics were used to summarize physician characteristics and the prevalence of sacred moments (experience and frequency) and how often they were discussed. Multivariable logistic regression was used to assess factors associated with experiencing sacred moments as well as the association of experiencing sacred moments with burnout manifestations. All models were adjusted for number of years practicing as an internal medicine physician, sex, and race. Two-sided *P* < .05 was considered statistically significant. Stata/SE, version 18.5 (StataCorp LLC) was used for all analyses.

## Results

A total of 629 of 1421 randomly selected internal medicine physicians completed a survey, resulting in a 44.3% response rate (Table 1). The median time participants had spent in practice was 23 (IQR, 15-29) years. Of the 617 respondents with data available, 237 (38.4%) were female, 376 (60.9%) were male, and 4 (0.6%) were other or preferred not to answer. Among the 611 respondents who provided data on race, 1 (0.2%) identified as American Indian or Alaska Native; 189 (30.9%), Asian; 31 (5.1%), Black or African American; 2 (0.3%), Native Hawaiian or Other Pacific Islander; 362 (59.2%), White; and 26 (4.3%), other; 29 (4.7%) of 611 identified as Hispanic or Latino. A total of 299 of 617 respondents (48.5 %) were hospitalists while 307 of 618 (49.7%) were primary care practitioners. A total of 424 of 626 respondents (67.7%) had experienced a sacred moment with a patient (Table 2). Experiencing sacred moments did not differ significantly between male (242 of 375 [64.5%]) and female (170 of 236 [72.0%]) physicians (*P* = .054); hospitalists (204 of 298 [68.5%]) and nonhospitalists (210 of 317 [66.2%]; *P* = .56); or inpatient (195 of 287 [67.9%]) and outpatient (194 of 289 [67.1%]) physicians (*P* = .83). The percentage of White physicians experiencing a sacred moment was significantly greater than physicians from other racial groups combined (263 of 362 [72.7%] vs 145 of 247 [58.7%]; *P* < .001). Number of years practicing as an internal medicine physician was not associated with experiencing a sacred moment (unadjusted odds ratio [OR], 1.00; 95% CI, 0.99-1.02; *P* = .74).

**Table 1.** Characteristics of Internal Medicine Physician Study Participants

Characteristic	Respondents, No./total No. (%)
Time spent in practice, median (IQR), y	23 (15-29)
Sex	
Female	237/617 (38.4)
Male	376/617 (60.9)
Other or prefer not to answer	4/617 (0.6)
Race	
American Indian or Alaska Native	1/611 (0.2)
Asian	189/611 (30.9)
Black or African American	31/611 (5.1)
Native Hawaiian or Other Pacific Islander	2/611 (0.3)
White	362/611 (59.2)
Other^a^	26/611 (4.3)
Hispanic or Latinx ethnicity	
Yes	29/611 (4.7)
No	582/611 (95.3)
Marital status	
Divorced	33/618 (5.3)
Married or living as if married	534/618 (86.4)
Separated	11/618 (1.8)
Single, never married	33/618 (5.3)
Widowed	7/618 (1.1)
Children younger than 18 y	
Yes	266/618 (43.0)
No	352/618 (57.0)
Clinical work setting	
Inpatient only	254/623 (40.8)
Outpatient only	236/623 (37.9)
Both inpatient and outpatient	100/623 (16.1)
Other	33/623 (5.3)
Type of facility for most clinical work	
Academic medical center or clinic	86/618 (13.9)
Community medical center or clinic	277/618 (44.8)
Veterans Affairs medical center or clinic	186/618 (30.1)
Other	69/618 (11.2)
Role^b^	
Hospitalist	299/617 (48.5)
Primary care practitioner	307/618 (49.7)
Time spent working per week, median (IQR), h	50 (40-70)

^a^ Includes anything that survey respondents chose to write in a free text question when they selected other: Afghan; Arab; Asian Indian; Egyptian; Hispanic; human; Indian; Iranian; Latino; Middle Eastern; Middle Eastern North African; Mediterranean; Mestizo; multiple races; Pakistani; Persian; Portuguese–African; South Asian; South Indian; Southeast Asian; White and American Indian or Alaskan Native; White and Asian; White and Black or African American; White, Black or African American, and American Indian or Alaskan Native; and White and Native Hawaiian or Other Pacific Islander.

^b^ These percentages do not equal 100%, because 2 separate dichotomous questions were posed: (1) Do you consider yourself a hospitalist? and (2) Do you provide primary care?

**Table 2.** Prevalence of and Perceptions About Sacred Moment Experiences

Sacred moment experience	Respondents, No./total No. (%)
Ever experienced a sacred moment with a patient	424/626 (67.7)
Frequency of experiencing a sacred moment with a patient	
Weekly	42/421 (10.0)
Monthly	60/421 (14.3)
A few times per year	146/421 (34.7)
Annually	18/421 (4.3)
A few times in my career	155/421 (36.8)
Frequency of discussing sacred moment experiences with colleagues	
Always	3/421 (0.7)
Often	16/421 (3.8)
Sometimes	88/421 (20.9)
Rarely	147/421 (34.9)
Never	167/421 (39.7)
“Experiencing a sacred moment with a patient helps me feel less burned out”	
Strongly agree	185/421 (43.9)
Somewhat agree	137/421 (32.5)
Neither agree nor disagree	78/421 (18.5)
Somewhat disagree	11/421 (2.6)
Strongly disagree	10/421 (2.4)

Of the 421 respondents who responded to questions regarding experience of a sacred moment, 42 (10.0%) experienced them weekly; 60 (14.3%), monthly; 146 (34.7%), a few times per year; 18 (4.3%), annually; and 155 (36.8%), a few times in their career. Of those who had experienced a sacred moment, most reported either never (167 [39.7%]) or rarely (147 [34.9%]) discussing them with colleagues. A total of 88 respondents (20.9%) reported discussing them sometimes; 16 (3.8%), often; and 3 (0.7%), always. Of those experiencing a sacred moment, 322 (76.5%) indicated that they agreed or strongly agreed that experiencing a sacred moment helped them feel less burned out.

Multivariable regression results highlighting factors found to have been associated with experiencing a sacred moment are presented in Table 3. Considering oneself a very spiritual person (OR, 2.23; 95% CI, 1.44-3.44; *P* < .001), having a strong sense of purpose in life (OR, 1.94; 95% CI, 1.36-2.76; *P* < .001) and work (OR, 1.94; 95% CI, 1.35-2.79; *P* < .001), believing that there is a life after death (OR, 1.81; 95% CI, 1.27-2.58; *P* = .001), praying in private at least once a week (OR, 1.83; 95% CI, 1.28-2.62; *P* = .001), and belief in God (OR, 1.80; 95% CI, 1.23-2.63; *P* = .002) were all associated with an approximate 2-fold increase in the odds of experiencing a sacred moment. Volunteering (OR, 1.52; 95% CI, 1.03-2.24; *P* = .03) and attending 4 or more in-person social events (OR, 1.53; 95% CI, 1.02-2.32; *P* = .04) within the last month were also associated with an increased odds of experiencing a sacred moment.

**Table 3.** Multivariable Associations Between Physician Characteristics and Sacred Moments^a^

Characteristic	OR (95% CI)	*P* value
Religious or spiritual beliefs		
Attend religious services at least once a month	1.66 (1.11-2.46)	.01
Pray privately at least once a week	1.83 (1.28-2.62)	.001
Believe in God	1.80 (1.23-2.63)	.002
Believe in life after death	1.81 (1.27-2.58)	.001
Consider oneself a very spiritual person	2.23 (1.44-3.44)	<.001
Sense of purpose		
Strong sense of purpose in life	1.94 (1.36-2.76)	<.001
Strong sense of purpose in work	1.94 (1.35-2.79)	<.001
Interpersonal connections		
Attending 4 or more in-person social events in the last month	1.53 (1.02-2.32)	.04
Volunteering in the last month	1.52 (1.03-2.24)	.03

Abbreviation: OR, odds ratio.

^a^ All models were adjusted for race, sex, and number of years practicing as an internal medicine physician.

A total of 382 of 622 respondents (61.4%) were classified as having at least 1 manifestation of burnout, whereas 61 of 622 (9.8%) met thresholds for all 3 burnout domains (emotional exhaustion, depersonalization, and reduced personal accomplishment) (Table 4). Any prior experience of a sacred moment was not associated with reduced odds of burnout (1 manifestation of burnout: OR, 0.95 [95% CI, 0.66- 1.36; *P* = .79]; extreme burnout: OR, 0.74 [95% CI, 0.42-1.31; *P* = .30]). However, those experiencing sacred moments a few times per year or more frequently had reduced odds of extreme burnout (OR, 0.29; 95% CI, 0.14-0.60; *P* = .001), compared with those experiencing sacred moments yearly or a few times in their career. Additionally, among those experiencing a sacred moment, discussing these moments with colleagues was found to be protective against burnout (1 manifestation of burnout: OR, 0.62 [95% CI, 0.40-0.95; *P* = .03]; extreme burnout: OR, 0.46 [95% CI, 0.23-0.94; *P* = .03]).

**Table 4.** Multivariable Associations Between Sacred Moments and Burnout^a^

Characteristic	Single manifestation of burnout		Extreme burnout	
	OR (95% CI)	*P* value	OR (95% CI)	*P* value
Ever experienced a sacred moment with a patient	0.95 (0.66-1.36)	.79	0.74 (0.42-1.31)	.30
Experienced a sacred moment with a patient a few times per year or more frequently	0.75 (0.49-1.15)	.19	0.29 (0.14-0.60)	.001
Discussed with colleagues about sacred moment experiences	0.62 (0.40-0.95)	.03	0.46 (0.23-0.94)	.03

Abbreviation: OR, odds ratio.

^a^ All models adjusted for race, sex, and number of years practicing as an internal medicine physician. Burnout outcomes are based on survey questions derived from the Maslach Burnout Inventory–Human Services Survey (MBI). One manifestation of burnout is defined as scoring high in the emotional exhaustion and/or depersonalization domains of the MBI. Extreme burnout is defined as scoring high in both the emotional exhaustion and depersonalization domains and low in the personal accomplishment domain of the MBI.

## Discussion

Internal medicine physicians commonly experience sacred moments with their patients, defined as deeply meaningful, memorable, and sometimes spiritual moments, as if time stood still. Despite the pervasiveness of sacred moments in medicine, our national survey found that physicians rarely discuss these meaningful experiences with their colleagues. This is noteworthy in the context of high rates of physician burnout. Our multivariable regression analysis found that while having ever experienced a sacred moment was not associated with reduced odds of burnout symptoms when analyzed dichotomously (yes or no), experiencing sacred moments more frequently than once per year was associated with reduced odds of extreme burnout compared with experiencing them less frequently, suggesting a dose response to the benefits of sacred moments. Additionally, discussing sacred moment experiences with colleagues was found to be associated with reduced odds of burnout.

We found many factors that were associated with experiencing a sacred moment. These factors can be categorized into 3 overarching themes: (1) spiritual or religious beliefs, including considering oneself a very spiritual person, belief in life after death, frequently praying in private, and belief in God; (2) purpose in life or work; and (3) connection with others, including volunteering and attending frequent in-person social events.

Sacred moments have been studied most extensively in the psychotherapeutic and the general community settings.^1,8^ We are aware of only 2 studies of sacred moments conducted in a sample including physicians.^2,4^ Our finding of highly prevalent sacred moment experiences among internal medicine physicians is consistent with the findings in both of these studies that included physicians in their samples.^2,4^ Quinn and colleagues^2^ conducted in-depth qualitative interviews with patients and health care workers and found that sacred moments were extremely common and generally regarded as deeply meaningful. Saint and colleagues^4^ conducted qualitative interviews of health care workers in a radiation oncology cohort, and in that study, most interviewees had also experienced a sacred moment.

We are not aware of previous studies among physicians looking at factors associated with sacred moments or association with burnout. However, in community samples, sacred moment experiences were found to be associated with religious or spiritual beliefs, interpersonal connections, openness to experience, and openness to touching on difficult, sensitive issues.^7,8^ Our survey results are consistent with these findings. Across all 3 studied settings—psychotherapy, general community, and medical—perceptions of sacred moments have been shown to be beneficial to all involved, positively impacting the overall wellness and stress levels of those who experienced these moments.^2,11,12^

Because of the extremely high rates of burnout among physicians, we were interested in studying whether sacred moment experiences could mitigate physician burnout. Potential hypothesized mechanisms included enhanced connection to others or greater meaning or purpose in life.^1,13^ While our multivariable regression results did not show an association between having ever experienced a sacred moment and burnout symptoms, those who experienced sacred moments more often were found to have reduced odds of extreme burnout compared with those who experienced them less often. This finding suggests a dose response to the benefits of sacred moments. Additionally, we found that discussing sacred moments was associated with lower odds of symptoms of burnout. This suggests that benefits of sacred moments may arise from the experience itself, the narrative formed about and around the experience, and the act of sharing the story with others.

Sacred moments are commonly experienced by internal medicine physicians and can leave participants with a sense of joy, peace, and empathy for the others involved. Identifying factors associated with sacred moments can inform interventions to foster these moments of deep connection between health care workers and patients. For example, more research could be conducted on whether physicians in certain specialties experience more sacred moments, or whether factors such as continuity of relationships with patients contribute to experiencing sacred moments. Additionally, future sacred moment research could be conducted comparing sacred moment experiences of physicians with other types of clinicians, such as nurses.

Creating a clinical milieu to make these moments more likely to occur and be discussed could decrease burnout rates among physicians. Potential interventions could include both system changes and individual interventions. System changes could include redesigning architectural clinical spaces in a way that promotes these connections (eg, accessible chair next to the patient’s bed for the clinician to talk to the patient at eye level, or ambient lighting and natural materials to promote a sense of calm), or system changes to support continuity of physician-patient relationships (eg, hospitalists following up with patients when they transfer to another unit) or to leave room for open-ended conversations within the clinical encounter. An example of an individual intervention is clinician training on how to foster these moments by creating space internally. Additionally, it may be beneficial to create time, space, and different venues for physicians to share their sacred moment experiences with each other. These venues could include dedicated conferences for sacred moment story sharing or platforms such as podcasts or websites devoted to the collection of sacred moments. Promoting awareness and discussions of these powerful connections between patients and clinicians could be protective against burnout among physicians, while simultaneously improving patient-centered care.

### Limitations

Our study has several limitations. First, the response rate to our survey was 44.3%, which may not reflect the entirety of all possible participants. However, this rate is greater than most rates achieved in other national surveys of physician respondents regarding burnout and wellness. Second, while we achieved representation from across the country, it is possible that respondents may differ from those physicians who chose not to participate in the study. Third, we only collected data on internal medicine physicians without a defined subspecialty. Assessing the degree to which experiencing sacred moments with patients varies by internal medicine subspecialty is an opportunity for future research. Additionally, given the cross-sectional design of our study, we could only show associations and not causation between experiencing sacred moments and physician characteristics including burnout. Last, it is possible that the associations detected between burnout and experiencing or talking about sacred moments could be mediated or confounded by other factors such as isolation or loneliness.^14,15^ This is another opportunity for future research.

## Conclusions

In this national survey measuring sacred moments among internal medicine physicians, we found that while sacred moments are common, discussing them with professional colleagues is rare. Multivariable analyses revealed that general internists who either experienced sacred moments often or frequently discussed them with colleagues had lower odds of reporting burnout. Given these potential benefits, understanding sacred moment experiences and encouraging discussion with professional colleagues should be encouraged.

## Notes

Supplement.
Data Sharing Statement
