# Supplementary material for: Sacred Moment Experiences Among Internal Medicine Physicians
Source: JAMA Netw Open. 2025 May 30;8(5):e2513159. doi: 10.1001/jamanetworkopen.2025.13159 (PMC12125638; doi:10.1001/jamanetworkopen.2025.13159)
Supplement: Supplement. — Data Sharing Statement [file jamanetwopen-e2513159-s001.pdf]

Data Sharing Statement

Ameling. Sacred Moment Experiences Among Internal Medicine Physicians. *JAMA Netw Open*. Published May 30, 2025. doi:10.1001/jamanetworkopen.2025.13159

Data
Data available: No
